# Morphological, immunohistochemical and molecular analysis of follicular dendritic cell sarcomas: L1CAM as a new diagnostic marker

**DOI:** 10.1111/his.15458

**Published:** 2025-04-27

**Authors:** Selina Schelbert, Katja Maurus, Sabine Roth, German Ott, Katrin S Kurz, Carolin Mogler, Barbara Wollenberg, John Linde, Alberto Zamo, Ioannis Anagnostopoulos, Susanne Gramlich, Andreas Rosenwald, Elena Gerhard‐Hartmann

**Affiliations:** ^1^ Institute of Pathology University of Würzburg Würzburg Germany; ^2^ Comprehensive Cancer Center Mainfranken Würzburg Germany; ^3^ Department of Clinical Pathology Robert‐Bosch‐Krankenhaus and Dr Margarete Fischer‐Bosch Institute of Clinical Pharmacology Stuttgart Germany; ^4^ Institute of Pathology, TUM School of Medicine and Health Technical University of Munich (TUM) Munich Germany; ^5^ Clinic for Otorhinolaryngology, Head and Neck Surgery MRI TUM, Technical University Munich Munich Germany

**Keywords:** CD171, FDC sarcoma, IHC, targeted genomic sequencing

## Abstract

**Aims:**

Follicular dendritic cell sarcoma (FDCS) is a rare neoplasm exhibiting morphological and immunophenotypical features of follicular dendritic cells. Given its rarity and broad morphological spectrum, diagnosis can be challenging. Knowledge of the molecular basis of this rare tumour is still limited. To further refine the biological and diagnostic characteristics of these neoplasms, we performed a comprehensive morphological, immunohistochemical and molecular analysis.

**Methods and results:**

As well as histopathological and immunohistochemical analysis, we performed molecular analysis by next‐generation panel sequencing of 15 tissue samples from 13 patients diagnosed with FDCS. In the histomorphological analysis of this FDCS series, we observed a morphological spectrum with a mixture of spindled and epithelioid cells (six of 13), but also cases with predominant epithelioid cytomorphology (seven of 13). We identified the L1 cell adhesion molecule (L1CAM) as a novel immunomarker of FDCS, as it was variably expressed in all cases. Sequencing led to the identification of 170 variants (classes 3, 4 and 5) in 112 genes. The most frequently detected (likely) pathogenic mutations affected *NFKBIA* (five of 13), leading to activation of nuclear factor kappa B (NFκB) signalling. Notably, deleterious *NFKBIA* mutations were only found in cases with predominant epithelioid morphology (five of seven). Furthermore, *TP53* mutations were detected in two cases with epithelioid morphology and high proliferation rate, and one of these cases relapsed twice.

**Conclusions:**

The morphological and genetic landscape of FDCS in this series was heterogeneous. However, in line with previous data, we identified recurrent genetic alterations affecting NFkB signalling. The expression of the adhesion molecule L1CAM might aid in the diagnosis of this uncommon neoplasia.

## Graphical abstract

Pathohistological analysis of FDCS revealed a morphological spectrum and led to the identification of the adhesion molecule L1CAM as a novel diagnostic immunomarker for FDCS. Sequencing identified NFKBIA as the most frequently altered gene, of note, exclusively in cases with a predominant epithelioid cytomorphology.

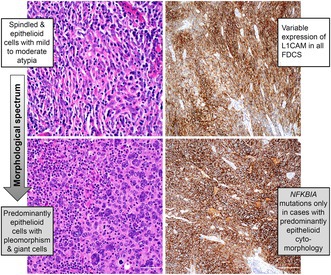

**Abbreviations**
CNVs: copy number variations
CR1: complement receptor CD35
CR2: complement receptor CD21
EBER‐1 and ‐2: EBV‐encoded RNAs
FCER2: Fc receptor CD23
FDC: follicular dendritic cell
FDCSP: FDC‐secreted protein
FFPE: formalin‐fixed, paraffin‐embedded
GC: germinal center
H&E: hematoxylin and eosin
hV‐CD: hyaline‐vascular variant of Castleman disease
ICAM1: intercellular adhesion molecule
IHC: immunhistochemistry
L1CAM: L1 cell adhesion molecule
LN: lymph node
MAPK: mitogen‐activated protein kinase
Mb: megabase
NFkB: nuclear factor kappa B
PAS: periodic acid‐Schiff
PD‐L1: programmed death ligand 1
SRGN: Serglycin
SSTR2a: somatostatin receptor 2a
TdT: terminal deoxynucleotidyl transferase
TMB: tumor mutational burden
VCAM1: vascular cell adhesion molecule
VUS: variants of uncertain significance

## Introduction

Follicular dendritic cell sarcoma (FDCS) is a rare neoplasm with morphological and immunophenotypical features of follicular dendritic cells (FDC).^1^ FDC are mesenchymal‐derived cells that have a crucial function in the germinal centre (GC) reaction by capturing, retaining and presenting antigens to B cells.^2^ FDC express adhesion molecules [such as vascular cell adhesion molecule 1 (VCAM1) and intercellular adhesion molecule (ICAM1)], which interact with their ligands on lymphocytes.^3^ Since the initial report of FDCS in 1986,^4^ several case reports and small case series have been published that have contributed to a more detailed characterisation of this peculiar tumour.^5^, ^6^ FDCS can arise in lymph nodes (LN) and at extranodal sites and usually exhibit a low to intermediate malignant but ultimately unpredictable behaviour. It can occur at any age, but most commonly affects adults (median age = 50 years), with a balanced gender ratio.^1^, ^6^ Microscopically, FDCS can exhibit a broad morphological spectrum and is occasionally associated with the hyaline‐vascular variant of Castleman disease (hv‐CD).^1^, ^7^, ^8^ Due to its various forms of appearance and rarity, FDCS is easily confused with other neoplasms.^5^ Currently, the diagnosis of FDCS relies upon morphology and immunohistochemistry (IHC), specifically upon the expression of classic FDC markers such as the complement/Fc receptors CD35 (CR1), CD21 (CR2) and CD23 (FCER2), which are involved in the antigen presentation by FDC.^1^, ^6^ Furthermore, a variety of other markers have been reported to be expressed in FDCS; e.g. Clusterin,^9^ CXCL13^10^ and, more recently, programmed death ligand 1 (PD‐L1) and somatostatin receptor 2a (SSTR2a),^11^ as well as FDC‐secreted protein (FDCSP) and Serglycin (SRGN).^12^ L1 cell adhesion molecule (L1CAM) is a cell adhesion glycoprotein that is involved in nervous system development.^13^, ^14^ Moreover, it has been described as a prognostic marker in endometrial cancer^15^, ^16^, ^17^ and other malignancies.^18^, ^19^, ^20^ However, it has been noticed that L1CAM is expressed in GC, specifically in FDC^21^, ^22^ and, like Horeweg *et al*.,^23^ we also observed L1CAM positivity in FDCs in lymphoid follicles in the surrounding of a *POLE*‐ultramutated endometrial cancer by IHC. This prompted us to investigate L1CAM expression in a group of reactive LNs and tonsils as well as in our series of FDCS.

Although some studies have performed molecular analysis of FDCS, knowledge of the molecular basis of this rare tumour is still limited. So far, no pathognomonic genetic alteration has been detected, but recurrent alterations affecting nuclear factor kappa B (NFκB) signalling, cell cycle control and tumour suppressor genes have been reported in FDCS.^24^, ^25^, ^26^, ^27^, ^28^

We performed a comprehensive morphological, immunohistochemical and molecular analysis of 13 FDCS cases, including one case that relapsed twice, to gain a more profound understanding of the pathobiological and diagnostic characteristics of FDCS.

## Materials and methods

### Case selection and evaluation

Fifteen tissue samples diagnosed as FDCS from 13 patients with sufficient formalin‐fixed, paraffin‐embedded (FFPE) tissue for further analyses were identified in the archives of the Lymphoma Reference Centers in Würzburg and Stuttgart, Germany, from 2015 to 2024. A histopathological review was conducted on haematoxylin and eosin (H&E) and immunostained slides according to the criteria of the 5th edition of the WHO Classification of Tumours of Haematopoietic and Lymphoid Tissues^1^ by expert haematopathologists (E.G.‐H., I.A., G.O., K.S.K., A.Z. and A.R.). Ethical approval was granted by the Local Ethics Committees.

### Histological and immunohistological methods

Histological sections (2 μm) were cut and stained with H&E, Giemsa and periodic acid‐Schiff (PAS) for routine histological evaluation. IHC was performed using FFPE tissue slides according to the manufacturers’ instructions and standard protocols on an automated immunostainer (BOND‐III, Leica Biosystems, Nussloch, Germany) (for details see Supporting information, Table S1).

### Epstein–Barr encoding region (EBER) *in‐situ* hybridisation

For detection of EBV‐encoded RNAs (EBER‐1 and ‐2), *in‐situ* hybridisation was performed on tissue sections using the Ventana ready‐to‐use kit according to the appropriate protocols on an automated immunostainer (Benchmark XT; Ventana/Roche, Munich, Germany).

### Next‐generation sequencing analysis

Genomic DNA and RNA were extracted from FFPE samples and processed with the commercially available TruSight Oncology 500 (TSO 500) DNA/RNA High‐Throughput Assay according to the TSO 500 High‐Throughput Assay protocol (Illumina, San Diego, CA, USA). Sequencing was performed on the NovaSeq 6000 (Illumina). Detailed information on the next‐generation sequencing analysis and data evaluation is provided in Supporting information, Data S1.

## Results

### Patients and sample characteristics

The series comprised 15 tissue samples from 13 patients diagnosed with FDCS, seven male and six female. One patient relapsed twice, and samples from all three time‐points were subjected to histopathological and molecular investigation. The median/mean patient age at diagnosis was 69/61.5 years (range = 15–83 years). Most of FDCS in this series (seven of 13, 58%) arose in LNs. Three cases (three of 13, 17%) occurred at extranodal sites, and an additional three cases affected both extranodal and nodal sites. Table 1 provides an overview of the clinicopathological characteristics. All cases (except for the relapsed) were sent to the Reference Centers to establish a primary diagnosis before therapy. Further clinical information, e.g. disease stage, follow‐up or treatment, was not available.

**Table 1:** Patient characteristics and pathomorphological features of FDCS

Case no.	Sex	Age at diag‐nosis (y)	Site	Size (cm)	Necrosis	Cytomorphologic features	Growth pattern	Special features
1	Female	23	Tonsil	NA	No	Mixed spindled and epithelioid tumour cells	Vaguely nodular	Partial infiltration
2	Male	15	LN (cervical)	3	No	Mixed spindled and epithelioid tumour cells	Vaguely nodular	Intermingled pleomorphic and giant cells, associated Castleman disease
3	Female	83	LN (cervical)	13.5	No	Mixed spindled and epithelioid tumour cells	Nodular	Accompanying sclerotic bands
4	Female	67	LN (cervical)	NA	Yes (focal)	Mixed spindled and epithelioid tumour cells	Vaguely nodular	Focally vessels with thickened hyaline walls
5	Female	32	LN (cervical), tonsil	4	No	Mixed spindled and epithelioid tumour cells	Vaguely nodular	Focal mild background sclerosis
6	Male	48	LN (abdominal) and liver	4 and 7 cm	No	Mixed spindled and epithelioid tumour cells	Vaguely nodular	Abundant B lymphocytes (no clonal B cell population)
7	Male	69	LN (cervical)	NA	No	Predominant epithelioid tumour cells	Diffuse/vaguely nodular	Many pleomorphic and multinucleated tumour cells
8	Male	82	LN (cervical)	NA	No	Predominant epithelioid tumour cells	Nodular	Focally vessels with thickened hyaline walls, intermingled pleomorphic and multinucleated tumour cells
9	Female	68	LN (cervical)	NA	No	Predominant epithelioid tumour cells	Patchy, serpiginous connected	No associated sclerosis/fibrosis
10	Male	74	Base of tongue	NA	No	Predominant epithelioid tumour cells	Diffuse/vaguely nodular	Focally vessels with thickened hyaline walls
11	Male	79	LN (lung hilus)	NA	Yes (focal)	Predominant epithelioid tumour cells	Vaguely nodular	Focal associated fibrosis/sclerosis, involved vessels
12	Female	79	Uterus	6	No	Predominant epithelioid tumour cells	Vaguely nodular	Accompanying hyaline sclerosis
13A	Male	80	LN (axilla)	NA	Yes (prominent)	Predominant epithelioid tumour cells	Vaguely nodular	Prominent necrosis, focal sclerotic bands
13B	Male	81	Upper arm (intramuscular)	NA	No	Predominant epithelioid tumour cells	Nodular	Many surrounding and intermixed lymphocytes and plasma cells, associated sclerotic bands
13C	Male	83	Axilla (soft tissue)	NA	No	Predominant epithelioid tumour cells	Intermixed and small clusters	Many surrounding and intermixed lymphocytes and plasma cells, background fibrosis/sclerosis

FDCS, follicular dendritic cell sarcoma; LN, lymph node; NA, not available; no., number; y, years.

### Histopathological findings

**Figure 1:**
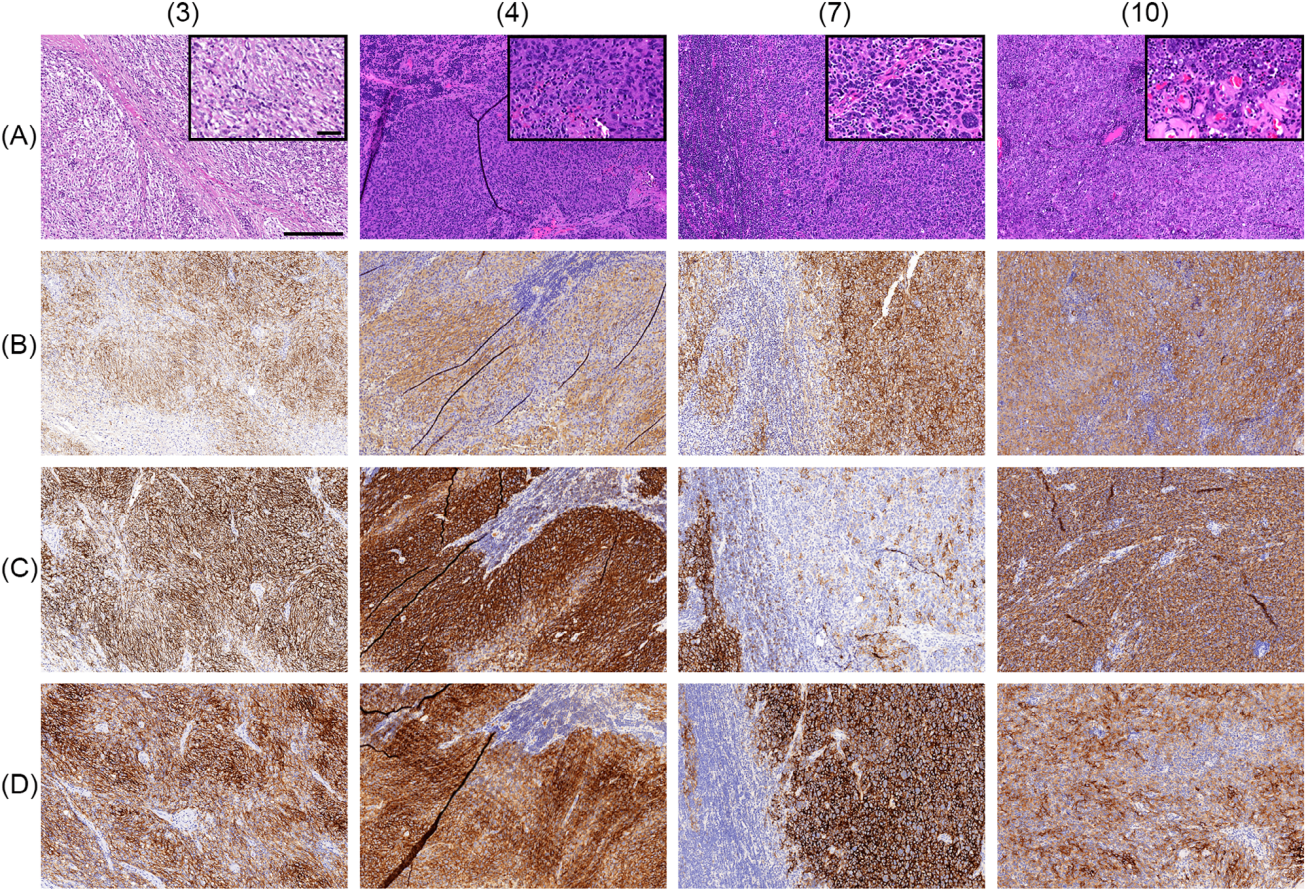
The morphological spectrum of follicular dendritic cell sarcoma (FDCS) and expression of L1 cell adhesion molecule (L1CAM). The histopathological spectrum (**A**, haematoxylin and eosin) of FDCS in our series ranges from with mixed spindled and epithelioid morphology to predominant epithelioid cells, sometimes with more atypia (cases 3, 4, 7 and 10). All cases expressed at least two of the three classic FDC markers CD21 (**B**), CD23 (**C**) and CD35 (not shown). All cases showed varying expression of L1CAM (**D**). The length of the scale bar is 250 μm, and the length of the scale bar of the magnified insert is 50 μm.

In line with the literature, a morphological spectrum was observed in our FDCS series. While six of 13 cases exhibited a mixture of spindled to oval and epithelioid tumour cells, seven of 13 showed a predominance of epithelioid tumour cells. Some cases displayed more cytological atypia with intermixed pleomorphic and giant cells (cases 2, 7 and 8), and three cases showed necrotic areas of variable extent (cases 4, 11 and 13A). The tumour cells were arranged in various, often mixed, growth patterns. However, in most cases a vaguely nodular pattern was predominant at low magnification. Occasionally, we observed associated sclerosis/fibrosis, and some cases showed focal thickened hyalinised blood vessels (cases 4, 8 and 10). A variable background infiltrate of lymphocytes, plasma cells and histiocytes was observed. In one case (case 6), manifesting in the liver and adjacent LNs, the neoplastic cells were accompanied by a prominent B cell infiltrate, which was suspicious of an indolent B cell lymphoma. However, immunophenotyping and lack of a clonal B cell population precluded the diagnosis of an (additional) B cell neoplasm. In case 2, FDCS was diagnosed in conjunction with hv‐CD. Interestingly, here we observed many accompanying terminal deoxynucleotidyl transferase (TdT)‐positive cells, while we found them only occasionally and in lower numbers in some of the other cases. In case 12 and in the relapsed cases (13B/C), a slight increase in EBER‐positive small lymphocytes was noticed, best suitable to a mild EBV‐reactivation, while the tumour cells were negative in the EBER *in‐situ* hybridisation. The histopathological findings are listed in Table 1 and exemplarily illustrated in Figure 1.

### Immunohistochemical analysis

Table 2 summarises the immunohistochemical findings in this FDCS series. All FDCS showed staining of at least two of the classic FDC markers CD21 (14 of 15 samples), CD23 (14 of 15 samples) and CD35 (14 of 15 samples), with variable intensity and ranging from focal to all tumour cells (Table 2, Figure 1). Furthermore, in all cases available for IHC we observed variable expression of PD‐L1 in the tumour cells which was, however, sometimes difficult to distinguish from the positivity of the associated immune cells, especially histiocytes. In 11 of 14 investigated cases, SSTR2A expression of various extent and intensity was observed, while D2‐40 expression was often only weak and/or partially/focally expressed in this series (not shown). However, the relatively modest expression of D2‐40 in this series might be due to the antibody clone used. The proliferation rate, determined by Ki67 IHC, ranged from 10 to 60%, with a median of 25%.

**Table 2:** Immunohistological features of FDCS

Case no.	CD21	CD23	CD35	L1CAM	D2‐40	PD‐L1	SSTR2a	P53 IHC	Tdt‐positive cells	Ki67 (%)
1	−/+ w	+/− v	−/+ v	+/− s	Negative	+/− v	−/+ v	NA	No	35
2	+ s	−/+ s	+v	+/− v	+v	+ v	Focal, w	Wt pattern	Many (focally > 100/HPF)	15
3	+v	+ s	+ v	+v	−/+ v	−/+ v	+/− w	Wt pattern	Occasionally (focally > 10 > 50/HPF)	10
4	+/− v	+ s	+ v	+ s	Focal, w	+/− v	+ v	Wt pattern	No	15
5	+ s	Negative	+ v	−/+ v	Focal, w	+ v	+/− v	Wt pattern	No	35
6	+/− v	+ s	p + (fix)	NA	−/+ v	NA	NA	NA	Occasionally (focally > 10 > 50/HPF)	20
7	+/− v	+/− v	−/+ v	+ s	Negative	−/+ v	+/− v	Wt pattern	No	25
8	−/+ v	+ s	+/− v	+ s	+/− v	−/+ v	+/− v	Wt pattern	NA	20
9	+ s	+/− v	+/− v	Focal, v	Negative	−/+ v (histiocytes!)	+ s	Wt pattern	Few (< 10/HPF)	20
10	+ v	+ s	+/− v (fix)	+/− v	+/− v	+ v	+ s	Wt pattern	No	20
11	−/+ v	+/− s	+/− v	+ s	−/+ w	+ s	+/− v	Mut/loss*	No	55
12	+/− v	+/− v	+ v	+/− v	Negative	−/+ v (histiocytes!)	Negative	Wt pattern	No	25
13A	Neg.	+/− s	+/− v	+/− s	Negative	+v	−/+ v	Mut/overexpression^*#*^	Focal, few (< 10/HPF)	40
13B	−/+ v	Focal, v	+/− v	−/+ v	Negative	+/− v	Negative	Mut/overexpression^#^	NA	60
13C	−/+ v	Focal, v	Negative	−/+ v	Focal, v	+/− v	Negative	Mut/overexpression^#^	Single	60

+: (nearly) all tumour cells positive; +/−: > 50% positive; −/+: < 50% positive.

FDCS, follicular dendritic cell sarcoma; Fix, fixation artefacts; mut, mutation; NA, not available; P+, peripherally positive; S, strong staining intensity; v, variable; w, weak; Wt, wild‐type.

* By panel sequencing, a TP53 frameshift mutation was detected (p.Tyr103LeufsTer44, AF: 30%).

^#^ By panel sequencing, a TP53 missense mutation was detected (p.Arg175His, missense, AF:19%, 13% and 12%, respectively, in 13A, B and C).

**Figure 2:**
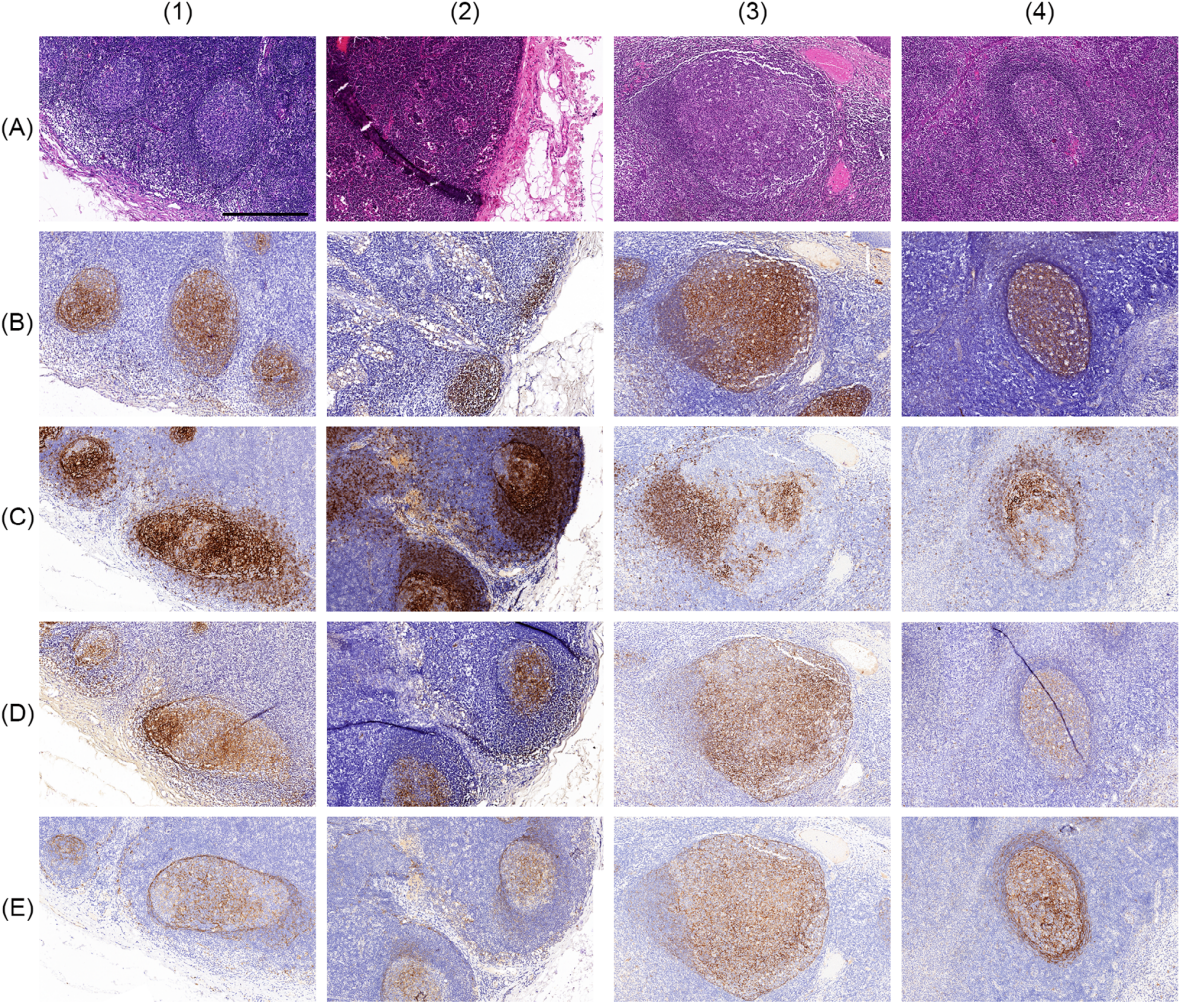
Expression of L1 cell adhesion molecule (L1CAM) in follicular dendritic cell (FDC) in the germinal centres of reactive lymphoid follicles. Haematoxylin and eosin (**A**) and immunohistochemical expression of CD21 (**B**), CD23 (**C**), CD35 (**D**) and L1CAM (**E**) in the germinal centres of lymphoid follicles in reactive lymph nodes (1, 2) and tonsils (3, 4). Length of the scale bar 500 μm.

In addition, we investigated the expression of L1CAM in a series of reactive LNs and tonsils. Here, we saw L1CAM expression corresponding to the FDC network (Figure 2). Furthermore, we performed L1CAM IHC in our FDCS series and observed L1CAM expression of varying extent and intensity in all cases. In 10 of 14 tested samples, L1CAM was positive in all or > 50% of the tumour cells (Table 2, Figure 1). Moreover, we investigated the expression of L1CAM in a series of 12 cases of mesenchymal and rare histiocytic neoplasms/proliferations, including four cases of histiocytic sarcoma, two cases of Rosai–Dorfman disease, one Langerhans cell histiocytosis, three cases of liposarcoma (including de‐differentiated and inflammatory liposarcoma) and one monophasic synovial sarcoma, as well as one tumour with features between an indeterminate dendritic cell tumour and a Langerhans cell sarcoma. All were negative for L1CAM.

### Molecular analysis

DNA and RNA were analysed by sequencing using the TSO 500 panel, and 14 samples from 12 patients were analysed at the Institute of Pathology, University of Würzburg. In this series, no oncogenic fusions could be detected. Furthermore, there was no evidence of microsatellite instability. The mean/median tumour mutational burden (TMB) (*n* = 12; two cases were excluded from this analysis due to substandard DNA quality) was 5.3 and 2.45 mutations per Megabase (Mb), respectively.

We detected a total of 170 variants in the 14 in‐house analysed samples, 49 of which were interpreted as (probably) oncogenic. Somatic variants detected by in‐house Illumina TruSight Oncology 500 panel sequencing are summarised in Supporting information, Tables S2–S4. The most frequently mutated gene in this series was *NFKBIA*. *NFKBIA* is a crucial inhibitor within the NFκB signalling pathway, which exhibited deleterious/loss of function mutations in five of 13 cases (38%). Intriguingly, *NFKBIA* mutations were only identified in cases displaying a predominantly epithelioid morphology (five of seven; 71% of patients in this subgroup).

**Figure 3:**
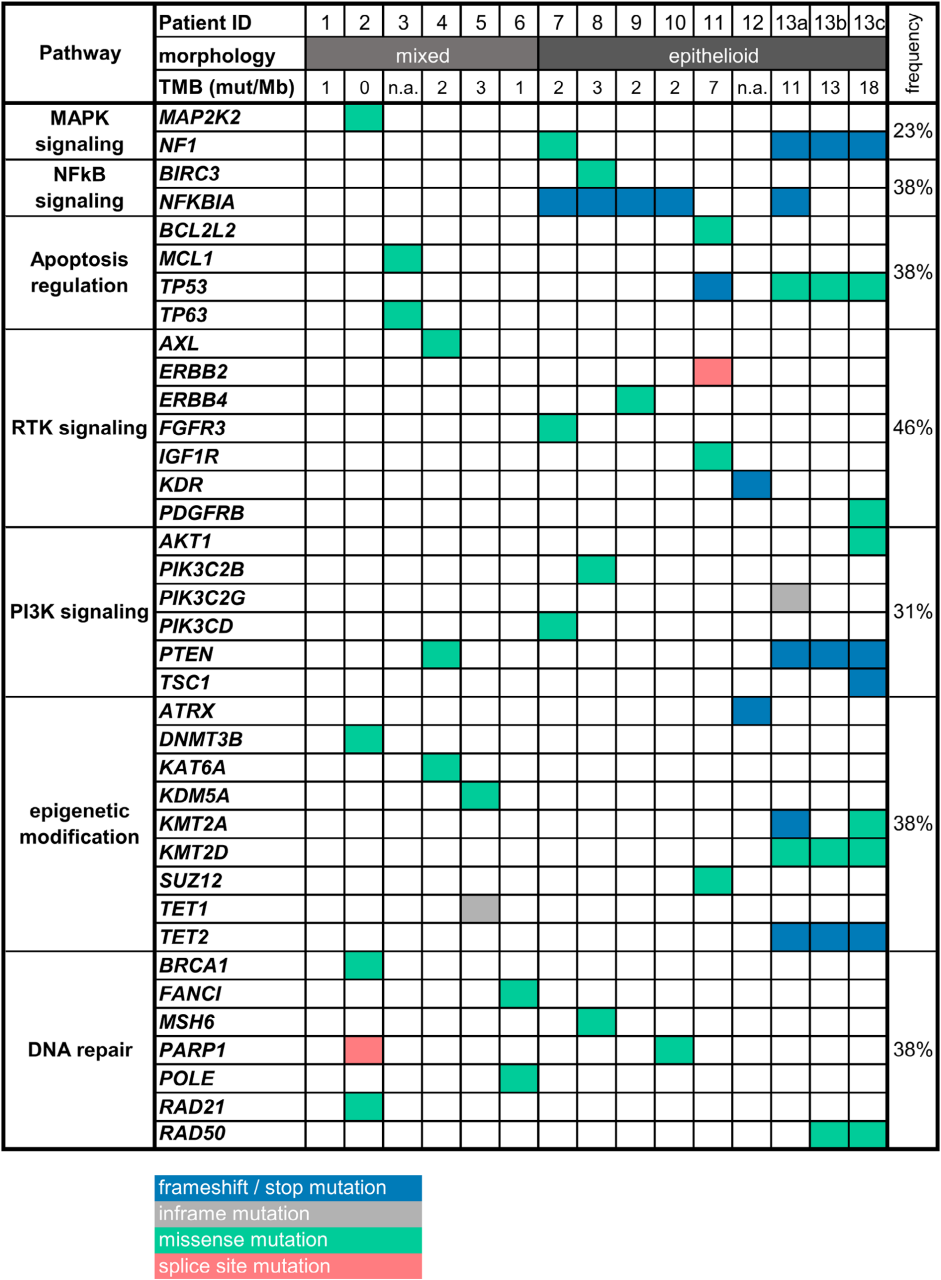
Mutation prevalence in follicular dendritic cell sarcoma (FDCS). Patients are mapped according to the morphology of their tumour (mixed spindled and epithelioid morphology and predominant epithelioid) and most frequent affected genes/pathways as well as tumour mutational burden (TMB) are displayed. N.a., not applicable.

Apart from this, the mutational spectrum in this FDCS series was somewhat heterogeneous. However, in individual cases, mutations affecting cellular processes such as epigenetic modification were discovered (*ATRX*, *KMT2A*, *KMT2D*, *TET2*). In addition, loss‐of‐function mutations have been identified in well‐described tumour suppressors that affect various signalling pathways, including cell cycle processes (*TP53*, *RB1*), PI3K/mTOR signalling pathway (*PTEN*, *TSC1*) and mitogen‐activated protein kinase (MAPK) signalling pathway (NF1). Figure 3 provides an overview of the genes/pathways most frequently affected in this FDCS series and the TMB. Looking at the copy number variations (CNVs), we also found a heterogeneous landscape. However, NFκB signalling was also affected by CNVs, with one homozygous deletion of *NFKBIA* and one homozygous deletion of *BIRC3*. In addition, we observed homozygous deletions of *CDKN2A* in two cases. Interestingly, we detected CNVs associated with MAPK signalling in two cases; namely, one *KRAS* and one *MAP2K1* amplification. No amplification of the PD‐L1/L2 gene locus was detected.

For case 1, sequencing was performed at another institution (at the Institute of Pathology, Technical University of Munich, also with the TSO 500 panel) and revealed two variants of unknown significance in *HSD3B1* and *ZFHX3*. Furthermore, there were indications of genomic gains in the *KDM6A* and *BTK* gene regions.

### Histopathological and molecular features of a relapsed FDCS at primary diagnosis and recurrence

This series includes one tumour that relapsed twice (case 13A/B/C). The initial resection (13A, 2018) of an affected axillary LN revealed a vaguely nodular infiltrate of predominant epithelioid tumour cells. Although this case did not exhibit prominent cytological atypia with pleomorphic and/or giant cells, as observed in other cases, it showed prominent necrosis. Extensive immunohistochemical analysis excluded a melanocytic or epithelial differentiation and identified expression of FDC typical immunomarkers (CD23 and CD35) and L1CAM. The tumour cells showed a relatively high proliferation rate of 40% by Ki67 IHC. Interestingly, p53 overexpression was detected by IHC, which was corroborated by identifying a pathogenic *TP53* mutation by panel sequencing. In addition, we detected oncogenic variants in *NF1*, *RB1*, *TET2*, *KMT2A*, *PTEN* and *NFKBIA*.

**Figure 4:**
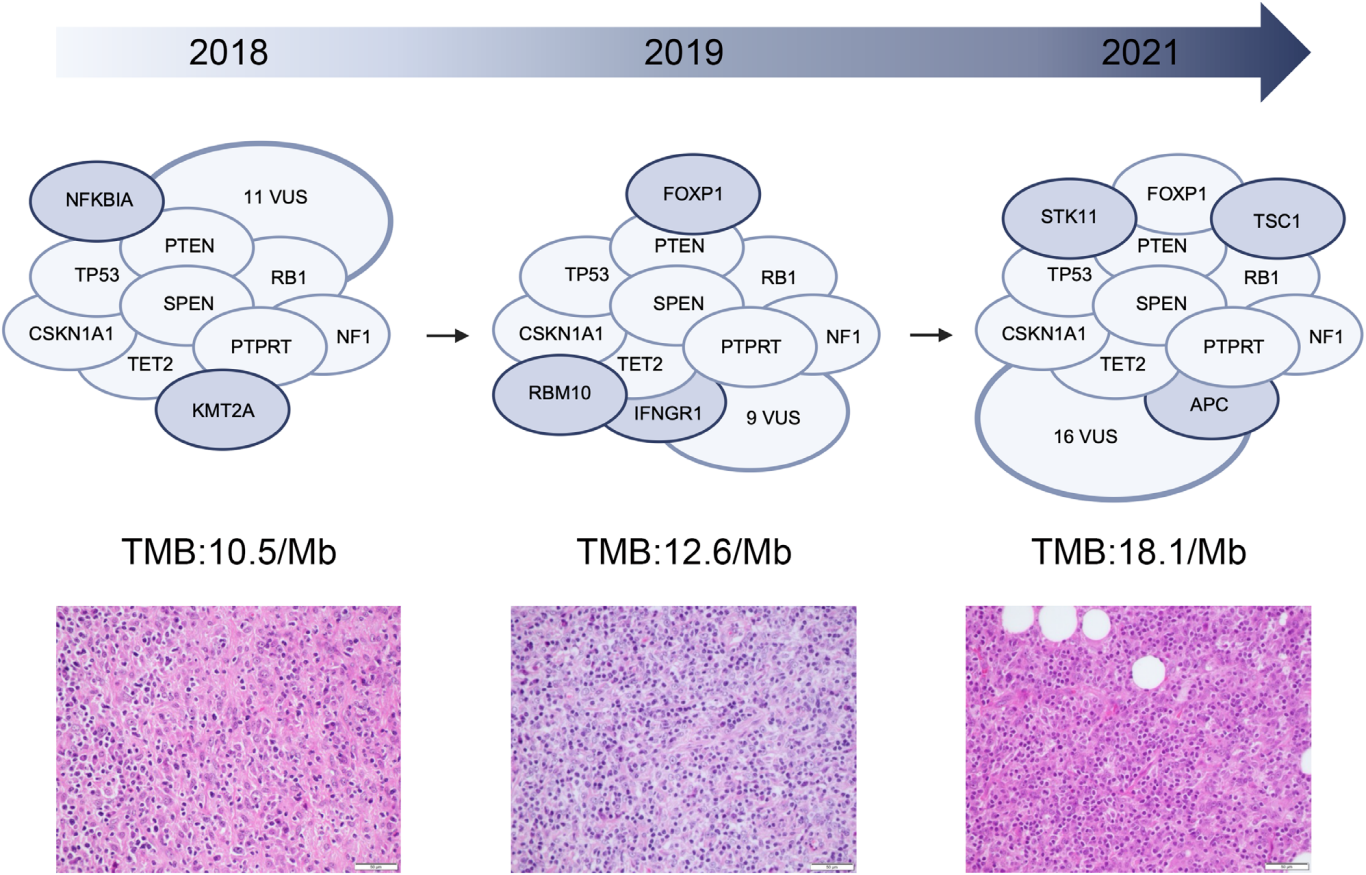
Mutational evolution and morphological features of a follicular dendritic cell sarcoma (FDCS) at primary diagnosis and relapse. Mutational evolution of a FDCS with predominant epithelioid morphology at primary diagnosis in 2018 (first column, case 13a), at first relapse in 2019 (second column, case 13b) and second relapse in 2021 (third column, case 13c) with increasing tumour mutational burden (TMB). The ellipses indicate genes carrying mutations that have been classified as likely pathogenic/pathogenic. The variants of uncertain significance (VUS) are only shown in number. The morphological features are shown in the haematoxylin and eosin images (length of the scale bar 50 μm,  lower row). The illustration with the ellipses was created in BioRender. Schelbert, S. (2025) https://BioRender.com/n52u496.

Sixteen months after the first resection, we received tissue from the upper arm with an intramuscular relapse of the FDCS (13B, 2019). Here, the tumour cells were accompanied by a more prominent lymphoplasmacytic background infiltrate. Seventeen months later, a second local recurrence was observed (13C, 2021), again with a relatively prominent inflammatory background. We also observed an increase in fibrosis which, however, may also be a consequence of therapeutic intervention (on which we have no further information). Using panel sequencing, several mutations in tumour suppressor genes were detected (unifying deleterious mutations in *TP53*, *PTEN*, *TET2*, *NF1* and *RB1*). We observed some differences in the mutation spectrum of the three tumour samples, probably reflecting clonal evolution and genetic tumour dynamics during disease. Interestingly, an initially detected *NFKBIA* mutation was lost at relapse, while an *APC* mutation was newly acquired. Moreover, this case presented the highest, and over time even increasing, TMB of this series in all three analysed specimens (10.5, 12.6 and 18.1 per Mb in 13A, 13B and 13C, respectively; mean/median TMB of all cases: 5.3/2.45 per Mb). Figure 4 gives an overview of the morphology and consecutive sequencing findings in the primary sample and at recurrence.

## Discussion

FDCS is a rare and diagnostically challenging neoplasm that can affect nodal and extranodal sites and may be confused with several other neoplasms. Currently, the diagnosis is based on the detection of morphological and immunohistochemical evidence of FDC differentiation. In line with the literature, we observed in our case series a morphological spectrum with different growth patterns and cytomorphological features, the latter ranging from mainly spindled to predominantly epithelioid tumour cells. As also well documented in the literature, some cases in this FDCS series showed intermixed pleomorphic and giant cells of various numbers. One case showed a very B cell‐rich background infiltrate, to the amount that a lymphoma was in differential diagnosis, and one case exhibited an association with hv‐CD. Given the considerable morphological diversity, the immunohistochemical findings supporting an FDC differentiation are paramount for the diagnosis.^1^

It has been noted that L1CAM is expressed in GC, specifically in FDC.^21^, ^22^ Like Horeweg and co‐workers,^23^ we also observed L1CAM expression in FDCs in lymphoid follicles surrounding a *POLE*‐ultramuted endometrial cancer. Therefore, we investigated the expression of L1CAM in a series of reactive LNs and tonsils and observed L1CAM expression corresponding to the FDC network in the GCs. As an adhesion glycoprotein, L1CAM is probably involved in antigen presentation by FDC. However, its exact biological functions and interactions in this setting are, at least to our knowledge, currently unclear. Interestingly, we also observed expression of L1CAM at various extents on all FDCS in this series, often consistently and strongly. In addition, we have investigated L1CAM expression in a small series of mesenchymal and histiocytic neoplasms/proliferations, which were all negative, suggesting that L1CAM expression may be used as a supportive marker for the diagnosis of FDCS. The fact that L1CAM is also expressed by non‐neoplastic FDC may be a hint that it is, in fact, biologically linked to this cell type, and not only a marker of aggressiveness, as reported for several types of epithelial cancer.^29^ It has been reported that the expression of L1CAM in pancreatic adenocarcinoma cells resulted in constitutive activation of NFkB,^30^ and L1CAM‐mediated signalling has been linked to NFkB activation.^31^ This is interesting, as the most common pathogenic genetic alterations in our series of FDCS also affect NFkB signalling, underlining the importance of this pathway in the pathogenesis of FDCS. However, further studies are needed to understand more clearly the biological functions of L1CAM in normal and neoplastic FDCs.

In line with the literature, the FDCS in our investigated cohort commonly expressed PD‐L1. However, regarding whether patients with recurrent/metastatic disease may benefit from immune check‐point inhibitors, data are limited to isolated case reports.^32^ Nevertheless, given the detected high TMB in one case that relapsed twice, this might be a reasonable option in selected cases. Also currently unclear is whether the frequently observed SSTR2a expression may offer theranostic options in these neoplasms.

In addition to morphological and immunohistochemical analyses, we performed panel sequencing in our FDCS series. In line with the results of Griffin *et al*.,^25^ the most obviously oncogenically altered pathway in the FDCS in our study was NFkB signalling, with deleterious mutations in *NFKBIA* being the most frequently affected gene (38%). In one additional case, there were indications for a homozygous *NFKBIA* deletion. NFKBIA is a negative regulator of NFkB signalling, and its inactivation in various haematological and solid malignancies suggests a tumour suppressor role.^33^, ^34^, ^35^, ^36^ While extranodal FDCS predominated in the series by Griffin and colleagues, here we observed *NFKBIA* mutations mainly in LN manifestations of FDCS, underlining a similar pathobiology in nodal and extranodal FDCS. Intriguingly, in our series *NFKBIA* mutations were only identified in cases with a predominant epithelioid cytomorphology (five of seven; 71% of patients in this subgroup). As recently reported by Lorenzi and co‐workers, we also commonly observed mutations in DNA damage repair‐related genes.^28^ Furthermore, *TP53* mutation and corresponding altered p53 immunohistochemical expression were observed in two of 13 FDCS in this series. These tumours displayed the highest proliferation rate determined by Ki67 and one of these cases relapsed twice, also suggesting unfavourable biological effects of TP53 mutations in this entity. Unlike that reported by a single previous study,^37^ but in line with others, we found no *BRAF* mutations in our FDCS series. However, in contrast to most other studies we detected occasional genetic alterations affecting other genes involved in MAPK signalling, such as MAP2K1 and NF1.^24^, ^26^ In contrast to many other sarcomas, we could not detect recurrent structural genomic changes/rearrangements in this FDCS series, which is in line with the findings from Lorenzi *et al*.^12^

## Conclusion

The morphological and genetic landscape of FDCS is heterogeneous. However, in line with previous data, we identified recurrent oncogenic genetic alterations affecting NFkB signalling; specifically, inactivation of *NFKBIA* as the most common genetic alteration. Moreover, we observed expression of the adhesion molecule L1CAM to various extents in all investigated FDCS and in FDC in reactive lymphoid follicles, which might aid in the diagnosis of this uncommon neoplasm.

## Conflicts of interest

The authors declare no conflicts of interest.

## Supporting information


**Data S1.** Supplementary Methods.
**Table S1.** Immunohistochemical antibody panel.
**Tables S2–S4.** Somatic variants (S2) and copy number variations (S3) detected by Illumina TruSight Oncology 500 panel sequencing as well as quality control parameters (S4).

## Data Availability

The data that support the findings of this study are available from the corresponding author upon reasonable request.
