# Supplementary material for: Morphological, immunohistochemical and molecular analysis of follicular dendritic cell sarcomas: L1CAM as a new diagnostic marker
Source: Histopathology. 2025 Apr 27;87(2):258–69. doi: 10.1111/his.15458 (PMC12232250; doi:10.1111/his.15458)
Supplement: Supplementary file 1 — Data S1. Supplementary Methods. Table S1. Immunohistochemical antibody panel. Tables S2–S4. Somatic variants (S2) and copy number variations (S3) detected by Illumina TruSight Oncology 500 panel sequencing as well as quality control parameters (S4). [file HIS-87-258-s001.zip › his15458-sup-0001-Supplementary_Methods_revised_changes_clean.docx]

**Supplementary Methods**

**DNA and RNA extraction**

The tumor cellularity ranged from 20% to 80 %. Genomic DNA and RNA were extracted (after microdissection, if applicable) using the Maxwell RSC Blood DNA Kit and the Maxwell RSC RNA FFPE Kit (Promega, Walldorf, Germany) on a Maxwell RSC 48 instrument. The extracted DNA and RNA were quantified using a Qubit 2.0 Fluorometer and the Qubit dsDNA/RNA Broad Range-Assay-Kits (ThermoFisher, Karlsruhe, Germany).

**Library Preparation and Sequencing**

DNA and RNA were processed with the TruSight Oncology 500 DNA/RNA High-Throughput Assay strictly according to the TruSight Oncology 500 High-Throughput Assay protocol (Illumina, San Diego, California, USA) starting with 150ng of DNA and 80ng of RNA. The protocol E TruSight Oncology 500 HT Library Denaturation and Dilution Method (Standard Loading) was used for library pooling. The pooled libraries were sequenced on a SP-Flow Cell on the NovaSeq 6000 (Illumina, San Diego, California, USA).

**Data evaluation**

*TruSight Oncology 500 analysis*

Sequencing data were processed using the Illumina DRAGEN TruSight Oncology 500 Analysis Software v2.1 on the Illumina DRAGEN Bio-IT Platform v4.0 with hg19 as reference genome. The variants reported in the CombinedVariantOutput.tsv were filtered using a custom python script. Read depths and population allele frequencies were read from the Annotated.json files. Only variants that passed all of the following filters were kept. Read depth of the alternate allele ≥ 10, alternate allele frequency ≥ 0.05, allele population frequency in gnomad_allAF, gnomadEx_allAF, 1000g_allAF < 0.002. Further, variants with certain consequence terms were removed as well as artifacts of the TruSight Oncology 500 panel that had been identified by the analysis of previous runs were excluded. Our laboratory's test series showed a limit of detection for variant calling of 0.03% allelic fraction.

Variants with the following consequence terms were removed from the output produced by the TSO500 Analysis Software:

intron variant

synonymous variant

3_prime_UTR_variant

5_prime_UTR_variant

downstream_gene_variant

upstream_gene_variant (except TERT)

Copy number variations were called using the DRAGEN TruSight Oncology 500 Analysis Software v2.5.3. Variants that were marked as duplication or deletion in the cnv.vcf-output-file were extracted and the copy number was calculated as suggested in the user guide. Amplifications were considered if the copy number was ≥ 5. Deletions were considered with a CN ≤ 1.2.

*In-house analysis pipeline*

The fastq- and bam-files produced by the DRAGEN TruSight Oncology 500 Analysis Software were used as input for variant-calling and fusion detection using an in-house pipeline. The read quality was checked using FastQC v 0.11.9 (28) and the raw reads were trimmed with Trim Galore 0.6.7 (<https://github.com/FelixKrueger/TrimGalore>). SNPs and indels were called using Mutect2 in GATK v 4.4.0.0 (29) followed by the FilterMutectCalls tool. Variants were annotated with the Ensembl Variant Effect Predictor v109 or v110 (30) and filtered using the same criteria as described in the previous section. Further, variants that were classified as ‚benign‘ or ‚likely_benign‘ in ClinVar as well as known artifacts were removed. Arriba v2.4.0 (31) was used to detect gene fusions.

Variants with the following consequence terms were kept in the output of the inhouse pipeline:

splice_acceptor-variant

splice_donor_variant

stop_gained

frameshift_variant

stop_lost

start_lost

inframe_insertion

inframe_deletion

missense_variant

splice_region_variant

splice_donor_region_variant

upstream_gene_variant (if TERT)

Conventional loss of function mutations (splice site mutations, indels and stop mutations) and variants with a clear ClinVar or oncoKB entry (pathogenic/oncogenic and likely pathogenic/oncogenic) were assessed as (likely) oncogenic.
