# Supplementary material for: Morphological, immunohistochemical and molecular analysis of follicular dendritic cell sarcomas: L1CAM as a new diagnostic marker
Source: Histopathology. 2025 Apr 27;87(2):258–69. doi: 10.1111/his.15458 (PMC12232250; doi:10.1111/his.15458)
Supplement: Supplementary file 1 — Data S1. Supplementary Methods. Table S1. Immunohistochemical antibody panel. Tables S2–S4. Somatic variants (S2) and copy number variations (S3) detected by Illumina TruSight Oncology 500 panel sequencing as well as quality control parameters (S4). [file HIS-87-258-s001.zip › his15458-sup-0002-Supplementary_Table_1_revised_changes_clean.docx]

**Supplementary Table 1: Immunohistochemical antibody panel.**

| Antibody | Supplier | Clone | Dilution |
| --- | --- | --- | --- |
| CD20 | DAKO/Agilent | L26 | 1:500 |
| CD21 | DAKO/Agilent | 1F8 | 1:200 |
| CD23 | Leica | 1B12 | 1:100 |
| CD35 | DAKO/Agilent | Ber-Mac-DRC | 1:50 |
| D2-40 | DAKO/Agilent | D2-40 | 1:400 |
| Ki-67 | DAKO/Agilent | MIB-1 | 1:200 |
| L1CAM | Epitomics | EP411 | 1:200 |
| PD-L1 | Cell Signalling | E1L3N | 1:200 |
| p53 | DAKO/Agilent | DO-7 | 1:100 |
| SSTR2a | Zytomed | polyclonal | 1:50 |
| TdT | DAKO/Agilent | EP266 | 1:50 |
